# Association of Oral Hypofunction with Frailty, Sarcopenia, and Mild Cognitive Impairment: A Cross-Sectional Study of Community-Dwelling Japanese Older Adults

**DOI:** 10.3390/jcm10081626

**Published:** 2021-04-12

**Authors:** Maya Nakamura, Tomofumi Hamada, Akihiko Tanaka, Keitaro Nishi, Kenichi Kume, Yuichi Goto, Mahiro Beppu, Hiroshi Hijioka, Yutaro Higashi, Hiroaki Tabata, Kazuki Mori, Yumiko Mishima, Yoshinori Uchino, Kouta Yamashiro, Yoshiaki Matsumura, Hyuma Makizako, Takuro Kubozono, Takayuki Tabira, Toshihiro Takenaka, Mitsuru Ohishi, Tsuyoshi Sugiura

**Affiliations:** 1 Department of Maxillofacial Diagnostic and Surgical Science, Field of Oral and Maxillofacial Rehabilitation, Graduate School of Medical and Dental Sciences, Kagoshima University, Kagoshima 890-0075, Japan; arimuramaya@gmail.com (M.N.); thamada@sagara.or.jp (T.H.); althair@dent.kagoshima-u.ac.jp (A.T.); k9296982@kadai.jp (K.N.); kkume@dent.kagoshima-u.ac.jp (K.K.); ygoto@dent.kagoshima-u.ac.jp (Y.G.); mbeppu@dent.kagoshima-u.ac.jp (M.B.); zio@dent.kagoshima-u.ac.jp (H.H.); k0970037@kadai.jp (Y.H.); k5258228@kadai.jp (H.T.); k4639792@kadai.jp (K.M.); k5782265@kadai.jp (Y.M.); k2309975@kadai.jp (Y.U.); k5124979@kadai.jp (K.Y.); k6917253@kadai.jp (Y.M.); 2 Department of Oral & Maxillofacial Surgery, Hakuaikai Medical Cooperation, Sagara Hospital, Kagoshima 892-0833, Japan; 3 Department of Physical Therapy, School of Health Sciences, Faculty of Medicine, Kagoshima University, Kagoshima 890-0075, Japan; makizako@health.nop.kagoshima-u.ac.jp; 4 Department of Cardiovascular Medicine and Hypertension, Graduate School of Medical and Dental Sciences, Kagoshima University, Kagoshima 890-0075, Japan; kubozono@m.kufm.kagoshima-u.ac.jp (T.K.); ohishi@m2.kufm.kagoshima-u.ac.jp (M.O.); 5 Department of Occupational Therapy, School of Health Sciences, Faculty of Medicine, Kagoshima University, Kagoshima 890-0085, Japan; tabitaka@health.nop.kagoshima-u.ac.jp; 6 Tarumizu Municipal Medical Center, Tarumizu Chuo Hospital, Kagoshima 891-2124, Japan; takenaka@tarumizumh.jp

**Keywords:** oral function, masticatory function, sarcopenia

## Abstract

Oral hypofunction is a new concept that addresses the oral function of older adults. Few studies have investigated the relationship between oral hypofunction and general health conditions such as frailty, sarcopenia, and mild cognitive impairment. This paper explores these relationships in a large-scale, cross-sectional cohort study. The relationships of oral hypofunction with frailty, sarcopenia, and mild cognitive impairment were examined using data from 832 individuals who participated in the 2018 health survey of the residents of Tarumizu City, Kagoshima Prefecture, Japan. Individuals with frailty, sarcopenia, and mild cognitive impairment had significantly higher rates of oral hypofunction. Frailty was independently associated with deterioration of the swallowing function (odds ratio 2.56; 95% confidence interval, 1.26–5.20), and mild cognitive impairment was independently associated with reduced occlusal force (odds ratio 1.48; 95% confidence interval, 1.05–2.08) and decreased tongue pressure (odds ratio 1.77; 95% confidence interval, 1.28–2.43). There was no independent association found between sarcopenia and oral function. In conclusion, early intervention for related factors such as deterioration of the swallowing function in frailty, reduced occlusal force, and decreased tongue pressure in mild cognitive impairment could lead to the prevention of general hypofunction in older adults.

## 1. Introduction

Japan’s population aging rate currently sits at 28.4%, and is expected to exceed 40% by 2060, making Japan an “aging advanced country” [1]. Japan’s population aging policies are gaining attention, as this phenomenon is expected to reach a global scale in the future. Increases in social security benefit claims, caregiver burden, and medical expenses resulting from the poor physical and cognitive functions that accompany aging are causes for concern. Early intervention for frailty and sarcopenia are integral for extending healthy life expectancy and improving quality of life, and are thought to curtail medical expenses as a result [2,3]. Thus, it is of extreme importance to study the onset mechanisms in the early stages of conditions such as frailty, sarcopenia, and mild cognitive impairment, and many studies and reports have pursued these objectives. Fairhall et al. [4] carried out a 12-month exercise intervention with frail subjects and reported a significant increase in walking speed in the intervention group compared to the nonintervention group. Based on a review of interventions for poor muscle strength and sarcopenia in older adults, Borst [5] asserted that resistance training is the most effective intervention for increasing strength and muscle mass in older adults. Karssemeijer et al. [6] reported that an intervention combining cognitive and physical exercise improved cognitive function in subjects with mild cognitive impairment or dementia. Further, as dysphagia and masticatory dysfunction are directly linked to malnutrition and ultimately lead to a poor general condition, it goes without saying that oral health and function impact physical function. Mann et al. [7] reported that dysphagia and masticatory dysfunction have a negative impact on nutritional intake in older adults. Maeda et al. [8] found that dysphagia was an independent risk factor for sarcopenia, while Murakami et al. [9] reported that decreased masticatory ability was significantly correlated with sarcopenia. In particular, poor oral function has been reported as a risk factor for frailty, sarcopenia, cognitive function, and even health-related quality of life (HRQOL) [10,11,12,13].

Currently, experts in Japan are assessing the status of oral function in older adults and attempting to improve “oral hypofunction,” the name for the condition in which there is poor overall oral function. “Oral hypofunction” is a new clinical concept proposed by the Japanese Society of Gerontology in 2016 which has been recognized by the Japanese insurance system since 2018 [14]. Oral hypofunction is not a structural condition, e.g., tooth loss or decay, but a functional, physiological condition comprising seven oral function indicators: poor oral hygiene, oral dryness, reduced occlusal force, decreased tongue-lip motor function, decreased tongue pressure, decreased masticatory function, and deterioration of swallowing function. Oral function in older adults is now assessed using this new concept, and evidence of a poor oral function is gradually accumulating. There are studies reporting that poor oral function such as tongue pressure, masticatory ability, and occlusal force was associated with frailty or sarcopenia [15,16,17,18,19]. However, few studies have analyzed the relationship between frailty and oral hypofunction using all evaluation criteria, and no studies have analyzed the relationship between oral hypofunction and mild cognitive impairment. Therefore, in this study, we carried out a cross-sectional statistical examination of the relationship between oral hypofunction and frailty, sarcopenia, and mild cognitive impairment using data from a 2018 prospective cohort study of ordinary residents of Tarumizu City, Kagoshima Prefecture, Japan.

## 2. Materials and Methods

### 2.1. Participants

This study used data from the 2018 Tarumizu Study. The Tarumizu Study is a prospective community cohort study of residents of Tarumizu City, Kagoshima Prefecture, Japan who are over the age of 40. The present study targeted adults aged 65 and over, and was conducted from June to December of 2018 with Kagoshima University Hospital, Tarumizu City Hall, and Tarumizu Chuo Hospital, collaborating to perform health checks. Residents who were hospitalized or lived at a care facility were excluded from participation. This study was approved by the Clinical Research Ethics Committee of Kagoshima University Hospital (ref no. 170351).

### 2.2. Diagnosis of Oral Hypofunction

Assessment and diagnosis of oral hypofunction were performed based on the 2016 diagnostic criteria of the Japanese Society of Gerontology with some modifications to the test methods. In short, we adopted the seven indicators, i.e., poor oral hygiene, oral dryness, reduced occlusal force, decreased tongue-lip motor function, decreased tongue pressure, decreased masticatory function, and deterioration of swallowing function and diagnosed oral hypofunction, if three or more of these indicators were assessed as having poor function [14]. Methods of testing for poor oral function are described below. All assessments were performed by a dentist who tested for oral hypofunction. In addition, oral and physical function tests were performed in different locations by different evaluators for appropriate blinding.

#### 2.2.1. Poor Oral Hygiene

Poor oral hygiene was assessed using the revised Tongue Coating Index (TCI) [20]. The tongue was divided into three sections (anterior, central, and posterior) and the degree of tongue coating was assessed by visual inspection. A diagnosis of poor oral hygiene was made when the TCI was 50% or higher.

#### 2.2.2. Oral Dryness

An oral moisture checker (Mucus, Life Co. Ltd., Saitama, Japan) was used to measure mucosal moistness of the central area of the tongue dorsum 1 cm from the apex. Three measurements were taken, and the median value was used. A diagnosis of oral dryness was made when the measured value was below 27.0.

#### 2.2.3. Reduced Occlusal Force

Miyaura et al. [21] and Ikebe et al. [22] reported that a reduction in the number of teeth reduces occlusal force and masticatory ability. In their review, Gotfredsen et al. [23] stated that oral function is generally well preserved if an individual retains at least 20 teeth. Based on these reports, the oral hypofunction diagnostic criteria stipulate that occlusal force can be assessed using either measurement of pressure indicating film or number of remaining teeth; the present study used the latter. A diagnosis of reduced occlusal force was made if the number of remaining teeth, excluding the remaining roots and teeth with mobility 3, was less than 20.

#### 2.2.4. Decreased Tongue-Lip Motor Function

Decreased tongue-lip motor function was assessed using oral diadochokinesis for the pronunciation of /pa/, /ta/, and /ka/ [24]. Participants were instructed to repeatedly pronounce each token as quickly as possible for five seconds, and the number of pronunciations was recorded using the pen-tapping method (in which the experimenter dots with a pen). A count of at least six pronunciations per second was recorded as good, and less than six was recorded as poor. A diagnosis of decreased tongue-lip motor function was made if even one token was recorded as poor.

#### 2.2.5. Decreased Tongue Pressure

Tongue pressure was measured using a tongue pressure measuring instrument (JMS tongue pressure measuring instrument TPM-01, JMS Co. Ltd., Hiroshima, Japan). Three measurements were taken, and the maximum value was used. A diagnosis of decreased tongue pressure was made if the measured value was less than 30 kPa.

#### 2.2.6. Decreased Masticatory Function

Decreased masticatory function was subjectively assessed using a questionnaire based on past research reports [10]. This assessment used a question with two potential answers: “are you usually able to, or not able to, chew tough foods?” A diagnosis of decreased masticatory function was made if the participant responded that they were not able to chew tough foods.

#### 2.2.7. Deterioration of Swallowing Function

The 10-item Eating Assessment Tool (EAT-10), proposed by the Japanese Society of Gerontology as a screening tool to assess oral function, was used to assess swallowing function. A score of 3 or above on this self-administered questionnaire indicates deterioration of swallowing function.

### 2.3. Evaluation of Frailty, Sarcopenia, and Mild Cognitive Impairment

Frailty was assessed using the five factors of exhaustion, slowness, poor muscle strength, low physical activity, and weight loss based on the phenotype model used in Fried et al.’s Cardiovascular Health Study [25]. Participants meeting three or more criteria were considered frailty. Sarcopenia was assessed using the 2016 evaluation criteria of the Asian Working Group for Sarcopenia [26,27]. The National Center for Geriatrics and Gerontology-Functional Assessment Tool was used to evaluate mild cognitive impairment [28]. All assessments were performed by well-trained assessors under supervising experienced physical therapists and occupational therapists.

### 2.4. Statistical Analysis

The relationships between oral hypofunction, frailty, sarcopenia, and mild cognitive impairment were explored using the chi-squared test. Multivariate analysis using binary logistic regression was performed to explore the effect of oral hypofunction on frailty, sarcopenia, and mild cognitive impairment. Age, gender, BMI, education level, and physical activity level were included as covariates of frailty and sarcopenia, while age, gender, BMI, and physical activity level were included as covariates of mild cognitive impairment. All analyses were performed using JMP (SAS Institute Inc., Cary, NC, USA). The standard for statistical significance was set at *p* < 0.05.

## 3. Results

### 3.1. Participant Characteristics

Participant characteristics are shown in Table 1. The average age of the participants was 74.9 ± 6.29, and the number of women was 529 (63.6%). Among all participants, assessment of the general conditions revealed 44 participants (5.5%) with frailty, 130 (16.4%) with sarcopenia, and 261 (31.4%) with mild cognitive impairment.

Oral hypofunction was present in 468 participants (56.3%). Concerning the individual diagnostic criteria for oral hypofunction, assessment results revealed 606 participants (72.8%) with poor oral hygiene, 374 (45.0%) with oral dryness, 447 (53.7%) with reduced occlusal force, 248 (29.8%) with decreased tongue-lip motor function, 302 (36.3%) with decreased tongue pressure, 161 (19.4%) with decreased masticatory function, and 138 (16.6%) with deterioration of swallowing function.

### 3.2. Oral Hypofunction Related to Frailty, Sarcopenia, and Mild Cognitive Impairment

The results for each diagnostic criterion of oral hypofunction in relation to frailty, sarcopenia, and mild cognitive impairment are shown in Table 2. The rates of oral hypofunction in participants with frailty, sarcopenia, and mild cognitive impairment were 79.6%, 73.1%, and 68.6%, respectively. These rates were higher than the rates of 54.6%, 52.9%, and 50.7%, respectively, for the healthy group, confirming significant relationships between frailty, sarcopenia, and mild cognitive impairment and rate of oral hypofunction.

#### 3.2.1. Poor oral Function and Frailty

Rates of reduced occlusal force, decreased tongue pressure, decreased masticatory function and deterioration of swallowing function in the frailty group were significantly higher than those in the healthy group at 75.0%, 52.3%, 31.8%, and 34.1%, respectively (Table 2).

#### 3.2.2. Poor oral Function and Sarcopenia

The rates of oral dryness, reduced occlusal force, decreased tongue-lip motor function, and decreased tongue pressure in the sarcopenia group were significantly higher than those in the healthy group at 53.9%, 70.0%, 37.7%, and 51.5%, respectively (Table 2).

#### 3.2.3. Poor Oral Function and Mild Cognitive Impairment

The rates of reduced occlusal force, decreased tongue-lip motor function, decreased tongue pressure, and decreased masticatory function in the mild cognitive impairment group were significantly higher than those in the healthy group at 64.8%, 37.6%, 48.3%, and 23.8%, respectively (Table 2).

### 3.3. Association with General Hypofunction and Oral Hypofunction in Multivariate Binary Logistic Analysis

Binary logistic regression was performed with frailty, sarcopenia, and mild cognitive impairment as the dependent variables and each indicator of oral hypofunction as the independent variables. The results are shown in Table 3.

#### 3.3.1. Poor Oral Function and Frailty

In crude models, frailty was significantly associated with reduced occlusal force, and deterioration of the swallowing function. Further, in adjusted models including age, gender, BMI, education level, and physical activity level as covariates, frailty was independently associated with deterioration of swallowing function (odds ratio 2.56; 95% confidence interval, 1.26–5.20) (Table 3).

#### 3.3.2. Poor Oral Function and Sarcopenia

In crude models, sarcopenia was significantly associated with oral dryness, reduced occlusal force, and decreased tongue pressure. In the adjusted models including age, gender, BMI, education level, and physical activity level as covariates, there was no independent association found between sarcopenia and oral function (Table 3).

#### 3.3.3. Poor Oral Function and Mild Cognitive Impairment

In crude models, mild cognitive impairment was significantly associated with oral dryness, reduced occlusal force, decreased tongue-lip motor function, and decreased tongue pressure. Further, in adjusted models including age, gender, BMI, and physical activity level as covariates, mild cognitive impairment was independently associated with reduced occlusal force (odds ratio 1.48; 95% confidence interval, 1.05–2.08) and decreased tongue pressure (odds ratio 1.77; 95% confidence interval, 1.28–2.43) (Table 3).

## 4. Discussion

Oral health is commonly said to impact general health [29].Poor individual oral functions such as tongue pressure or oral diadochokinesis have previously been reported to lead to poor general condition [11,30]. Thus, although it may be important to prevent poor oral function in order to prevent frailty and sarcopenia, there is no clear evidence for this. At the same time, in 2016, the Japanese Society of Gerontology integrated the aforementioned indicators of poor oral function to define a new concept: oral hypofunction [14]. There are insufficient reports on the relationship between poor general function, i.e., frailty, sarcopenia, and mild cognitive impairment, and oral hypofunction. Thus, this study investigated the relationship of oral hypofunction with frailty, sarcopenia, and mild cognitive impairment—conditions representative of poor general function—in a large-scale cohort. The cohort used in this study was from Tarumizu City in Kagoshima Prefecture. Tarumizu City has a population of 14,000 with a population aging rate of 40% and no marked influx or efflux of residents. As such, the city was selected as a model community approximating the estimated population aging rate of 40% expected in Japan by 2060.

Previous studies of oral hypofunction in other Japanese cohorts aged 65 and older have found differing rates of oral hypofunction. Shimazaki et al. [31] found that the rate of oral hypofunction in adults aged 65 and older was 60% in a cohort with a 20% aging rate and a mean age of 73.2 for men and 72.9 for women, and Kugimiya et al. [32] reported a rate of 43.6% in a cohort with a 38% aging rate and a mean age of 76.3 ± 6.5. The present study had a mean age of 74.9 ± 6.29 and found an oral hypofunction rate of 56.3%. These results may be due to the fact that a community’s aging rate or mean age has little relation to the onset of poor oral function in older adults. Rather, there are differences in the characteristics of the older adults living in the community—for example, unique traits of people in the region, education, disease prevalence, frequency of dental care, and interest in oral hygiene—that impact the onset of poor oral function of older adults. In fact, the Tarumizu Study revealed that the rate of dental clinic visits within the past year, frequency of toothbrushing per day, number of oral medications, and education level were all associated with oral hypofunction. These associations require follow-up as a research topic in the future. The methods for assessment of oral function also differed between the present study and previous studies. It is thus considered essential to carry out a longitudinal study within the same cohort over time, or a nationwide cross-sectional study using the same research platform.

Frailty, sarcopenia, and mild cognitive impairment have been found to increase with age. While it has also been clarified that poor oral function similarly appears with age, the relationship between poor general function and poor oral function remains unclear. This study found significantly higher rates of oral hypofunction in the frailty, sarcopenia, and mild cognitive impairment groups. Reduced occlusal force, decreased tongue pressure, decreased masticatory function, and deterioration of the swallowing function were significantly more common in the frailty group, a result that matches that of past research. Meanwhile, existing reports on the relationship between sarcopenia and occlusal force assessed using number of teeth or pressure indicating film have not yielded consistent results [33]. For example, in two directly contradictory reports, Tanaka et al. [10] found a significant relationship between the number of teeth and sarcopenia, but no significant difference in the relationship between sarcopenia and occlusal force measured via pressure-indicating film. However, Murakami et al. [9] found no significant relationship between the number of teeth and sarcopenia, but did find a significant difference in the relationship between sarcopenia and occlusal force measured via pressure indicating film. A separate study by Horibe et al. [34] found no significant relationship between reduction in occlusal force and the sarcopenia evaluation items of grip strength and skeletal muscle index (SMI). Our research found significantly higher rates in the sarcopenia group, not only for reduced occlusal force—as measured by the number of teeth—but also for oral dryness, decreased tongue-lip motor function, and decreased tongue pressure. With the exclusion of oral dryness, all of these factors are thought to reflect a poor muscle strength, indicating that a poor muscle strength appears in much the same way in the oral context.

There have been a few studies showing that cognitive function was associated with poor oral hygiene and occlusal force assessment using the number of teeth. A recent meta-analysis [35] reported that masticatory dysfunction was positively associated with an increased risk of cognitive deficit, while Fukushima et al. [36] reported in animal studies that the reduction in masticatory stimuli affect memory and learning function. However, dementia patients struggle to follow test directions, making it difficult to determine proper results or to perform objective measurement using machines. Many studies have reported that cognitive function improved through dental care interventions that enabled patients with cognitive impairment to chew and eat. This is being used as a basis for the hypothesis that a poor mastication and feeding functions causes a poor cognitive function. However, while there are studies on the relationship between mild cognitive impairment and individual oral functions, there are no studies on its relationship to oral hypofunction. Delwel et al. [37] reported that masticatory function and swallowing function in older adults with mild cognitive impairment were good, but their evaluation used a subjective questionnaire assessment and no objective testing. Studies using objective functional evaluations found that decreased tongue-lip motor function assessed using oral diadochokinesis led to poor cognitive function (Watanabe et al. [38]), and that both tongue pressure and oral diadochokinesis can lead to poor cognitive function (Kugimiya et al. [11]). However, the method for evaluating mild cognitive impairment differed in these studies, which used the Mini-Mental State Examination, compared to the present study, which used the National Center for Geriatrics and Gerontology Functional Assessment Tool. This study focused on mild cognitive impairment and oral hypofunction and a comprehensive evaluation of oral function. Oral hypofunction was significantly higher in the group with mild cognitive impairment. In particular, the rates of concurrent reduced occlusal force, decreased tongue-lip motor function, decreased tongue pressure, and decreased masticatory function were significantly higher. In other words, poor function occurred despite the test of oral function being properly conducted, as participants in the mild cognitive impairment group were able to follow the test directions. This indirectly supports the previously-mentioned hypothesis that a poor mastication and feeding functions causes a poor cognitive function. Thus, it was revealed in the present study that oral hypofunction was associated with frailty, sarcopenia, and mild cognitive impairment.

We conducted a multivariate analysis using binary logistic regression to examine which of the seven indicators of oral hypofunction were more associated with frailty, sarcopenia, and mild cognitive impairment. The results demonstrated that frailty was independently associated with deterioration of the swallowing function, and mild cognitive impairment was independently associated with reduced occlusal force and decreased tongue pressure. However, although a chi-square test showed significant differences in the rates of poor oral function in the sarcopenia group, sarcopenia was not found to be independently associated with oral function in the multivariate analysis. Possible reasons for the above are, because sarcopenia is a physical disorder arising due to the deterioration of muscle, other factors such as age and nutrition may be more significant risk factors than poor oral function.

This study targeted people who could walk and participate in the medical examination. Therefore, residents who were hospitalized or lived at a care facility were excluded; had these people participated, it is very likely that the number of residents evaluated as having poor functions would have increased. In order to target all people living in the area, evaluators should go to aged-care facilities, which is an issue for our cohort in the future.

As this was a cross-sectional study using only data from 2018, a longitudinal study over time is essential to determine to what extent improvement in oral hypofunction can be achieved through dental care interventions. To evaluate the conclusions suggested by this research, we plan to divide participants into groups with and without oral hypofunction and explore the onset rates of frailty, sarcopenia, and mild cognitive impairment over time. In this study, we used simple tests of poor oral hygiene, occlusal force, and masticatory function in order to reduce the physical burden on participants. It is essential to use the same tests going forward to enable comparison with other cohorts. Thus, a consistent testing method is considered another challenge for future studies.

## 5. Conclusions

In conclusion, in this study, we investigated the relationship of oral hypofunction with the seven indicators of poor oral function, as well as frailty, sarcopenia, and mild cognitive impairment. This is the first such study on the relationship of oral hypofunction with sarcopenia and mild cognitive impairment. Our results suggest that it may be possible to prevent or improve frailty, sarcopenia, and mild cognitive impairment by preventing, delaying, or improving oral hypofunction. Simply improving oral hypofunction perhaps tackles the problem when is it already too late. Thus, different avenues, from prevention to therapeutic interventions, should be pursued.

## Figures and Tables

**Table 1:** Characteristics of the Participants.

Characteristic	M ± SD
Age (years)	74.9 ± 6.29
	*n*^2^ (%)
Men	303 (36.4)
Women	529 (63.6)
Physical hypofunction	
Frailty (*n*^1^ = 804)	44 (5.5)
Sarcopenia (*n*^1^ = 795)	130 (16.4)
Mild cognitive impairment (*n*^1^ = 831)	261 (31.4)
Oral hypofunction (*n*^1^ = 832)	468 (56.3)
Poor oral hygiene	606 (72.8)
Oral dryness	374 (45.0)
Reduced occlusal force	447 (53.7)
Decreased tongue-lip motor function	248 (29.8)
Decreased tongue pressure	302 (36.3)
Decreased masticatory function	161 (19.4)
Deterioration of swallowing function	138 (16.6)

Note. M, median; SD, standard deviation; *n*^1^, total number; *n*^2^, applicable number.

**Table 2:** Association of Oral Hypofunction with Frailty, Sarcopenia, and Mild Cognitive Impairment.

		**Frailty**		
	**Score**	**Nonfrailty**	**Frailty**	***p***
Oral hypofunction	<3	345 (45.4%)	9 (20.4%)	0.001
	≥3	415 (54.6%)	35 (79.6%)	
Poor oral hygiene	<50	211 (27.8%)	10 (22.7%)	0.467
	≥ 50	549 (72.2%)	34 (77.3%)	
Oral dryness	≥27	422 (55.5%)	19 (43.2%)	0.110
	<27	338 (44.5%)	25 (56.8%)	
Reduced occlusal force	≥20	362 (47.6%)	11 (25.0%)	0.003
	<20	398 (52.4%)	33 (75.0%)	
Decreased tongue-lip motor function	good	539 (70.9%)	27 (61.4%)	0.177
	poor	221 (29.1%)	17 (38.6%)	
Decreased tongue pressure	≥ 30	492 (64.7%)	21 (47.7%)	0.022
	<30	268 (35.3%)	23 (52.3%)	
Decreased masticatory function	good	619 (81.5%)	30 (68.2%)	0.030
	poor	141 (18.5%)	14 (31.8%)	
Deterioration of swallowing function	<3	640 (84.2%)	29 (65.9%)	0.002
	≥3	120 (15.8%)	15 (34.1%)	
		**Sarcopenia**		
	**Score**	**Nonsarcopenia**	**Sarcopenia**	***p***
Oral hypofunction	<3	313 (47.1%)	35 (26.9%)	<0.0001
	≥3	352 (52.9%)	95 (73.1%)	
Poor oral hygiene	<50	181 (27.2%)	36 (27.7%)	0.912
	≥50	484 (72.8%)	94 (72.3%)	
Oral dryness	≥27	372 (55.9%)	60 (46.1%)	0.041
	<27	293 (44.1%)	70 (53.9%)	
Reduced occlusal force	≥20	332 (49.9%)	39 (30.0%)	<.0001
	<20	333 (50.1%)	91 (70.0%)	
Decreased tongue-lip motor function	good	478 (71.9%)	81 (62.3%)	0.029
	poor	187 (28.1%)	49 (37.7%)	
Decreased tongue pressure	≥30	447 (67.2%)	63 (48.5%)	<0.0001
	<30	218 (32.8%)	67 (51.5%)	
Decreased masticatory function	good	542 (81.5%)	100 (76.9%)	0.226
	poor	123 (18.5%)	30 (23.1%)	
Deterioration of swallowing function	<3	560 (84.2%)	101 (77.7%)	0.069
	≥3	105 (15.8%)	29 (22.3%)	
		** Mild Cognitive Impairment (MCI) **		
	** Score **	** Non-MCI **	** MCI **	*** p ***
Oral hypofunction	<3	281 (49.3%)	82 (31.4%)	<0.0001
	≥3	289 (50.7%)	179 (68.6%)	
Poor oral hygiene	<50	154 (27.0%)	71 (27.2%)	0.956
	≥50	416 (73.0%)	190 (72.8%)	
Oral dryness	≥27	327 (57.4%)	131 (50.2%)	0.054
	<27	243 (42.6%)	130 (49.8%)	
Reduced occlusal force	≥20	292 (51.2%)	92 (35.2%)	<0.0001
	<20	278 (48.8%)	169 (64.8%)	
Decreased tongue-lip motor function	good	420 (73.7%)	163 (62.4%)	0.001
	poor	150 (26.3%)	98 (37.6%)	
Decreased tongue pressure	≥30	394 (69.1%)	135 (51.7%)	<0.0001
	<30	176 (30.9%)	126 (48.3%)	
Decreased masticatory function	good	471 (82.6%)	199 (76.2%)	0.031
	poor	99 (17.4%)	62 (23.8%)	
Deterioration of swallowing function	<3	480 (84.2%)	214 (82.0%)	0.424
	≥3	90 (15.8%)	47 (18.0%)	

**Table 3:** Odds Ratios for Frailty, Sarcopenia, and Mild Cognitive Impairment According to Oral Hypofunction.

		**Frailty**			
	**Score**	**Crude OR (95% CI)**	***p***	**^a^ Adjusted OR (95% CI)**	***p***
Poor oral hygiene	<50	1	0.52	1	0.85
	≥50	1.28 (0.61–2.68)		1.08 (0.49–2.36)	
Oral dryness	≥27	1	0.11	1	0.11
	<27	1.67 (0.89–3.11)		1.72 (0.89–3.31)	
Reduced occlusal force	≥20	1	0.03	1	0.24
	<20	2.28 (1.10–4.71)		1.61 (0.73–3.55)	
Decreased tongue-lip motor function	good	1	0.52	1	0.37
	poor	1.24 (0.65–2.37)		0.72 (0.35–1.48)	
Decreased tongue pressure	≥30	1	0.08	1	0.31
	<30	1.76 (0.94–3.30)		1.41 (0.73–2.74)	
Decreased masticatory function	good	1	0.24	1	0.26
	poor	1.51 (0.76–3.01)		1.52 (0.73–3.16)	
Deterioration of swallowing function	<3	1	0.01	1	0.009
	≥3	2.37 (1.21–4.63)		2.56 (1.26–5.20)	
		**Sarcopenia**			
	**Score**	**Crude OR (95% CI) **	***p***	**^a^ Adjusted OR (95% CI)**	***p***
Poor oral hygiene	<50	1	0.77	1	0.71
	≥50	0.94 (0.61–1.45)		0.91 (0.56–1.49)	
Oral dryness	≥27	1	0.03	1	0.16
	<27	1.54 (1.04–2.27)		1.37 (0.89–2.11)	
Reduced occlusal force	≥20	1	0.0003	1	0.19
	<20	2.17 (1.42–3.31)		1.39 (0.85–2.28)	
Decreased tongue-lip motor function	good	1	0.27	1	0.18
	poor	1.26 (0.84–1.90)		0.72 (0.44–1.16)	
Decreased tongue pressure	≥ 30	1	0.0004	1	0.22
	<30	2.03 (1.38–3.00)		1.32 (0.85–2.06)	
Decreased masticatory function	good	1	0.96	1	0.75
	poor	1.01 (0.63–1.63)		0.92 (0.53–1.57)	
Deterioration of swallowing function	<3	1	0.23	1	0.26
	≥ 3	1.34 (0.83–2.16)		1.35 (0.80–2.30)	
		** Mild cognitive impairment **			
	**Score**	**Crude** **OR (95% CI)**	***p***	**^b^Adjusted OR (95% CI)**	***p***
Poor oral hygiene	<50	1	0.79	1	0.51
	≥ 50	0.95 (0.68–1.34)		0.89 (0.62–1.26)	
Oral dryness	≥ 27	1	0.047	1	0.10
	<27	1.36 (1.00–1.84)		1.30 (0.95–1.78)	
Reduced occlusal force	≥ 20	1	0.0009	1	0.02
	<20	1.71 (1.25–2.35)		1.48 (1.05–2.08)	
Decreased tongue-lip motor function	good	1	0.02	1	0.36
	poor	1.46 (1.05–2.01)		1.17 (0.83–1.66)	
Decreased tongue pressure	≥ 30	1	<0.0001	1	0.0005
	<30	1.98 (1.45–2.70)		1.77 (1.28–2.43)	
Decreased masticatory function	good	1	0.27	1	0.28
	poor	1.24 (0.85–1.80)		1.24 (0.84–1.83)	
Deterioration of swallowing function	<3	1	0.99	1	0.95
	≥ 3	1.00 (0.67–1.49)		0.98 (0.65–1.49)	

Note. OR, odds ratio; CI, confidence interval. ^a^ Adjusted for age, gender, body mass index, education, and physical activity levels. ^b^ Adjusted for age, gender, body mass index, and physical activity levels.

## Data Availability

The data presented in this study are available on request from the corresponding author.
